# Efficacy of “Attachment-Based Compassion Therapy” in the Treatment of Fibromyalgia: A Randomized Controlled Trial

**DOI:** 10.3389/fpsyt.2017.00307

**Published:** 2018-01-16

**Authors:** Jesús Montero-Marín, Mayte Navarro-Gil, Marta Puebla-Guedea, Juan V. Luciano, William Van Gordon, Edo Shonin, Javier García-Campayo

**Affiliations:** ^1^Primary Care Prevention and Health Promotion Research Network (RedIAPP), Zaragoza, Spain; ^2^Faculty of Social and Human Sciences, Department of Psychology and Sociology, University of Zaragoza, Zaragoza, Spain; ^3^Teaching Research & Innovation Unit, Parc Sanitari Sant Joan de Déu, St Boi de Llobregat, Spain; ^4^Primary Care Prevention and Health Promotion Research Network, RedIAPP, Madrid, Spain; ^5^Centre for Psychological Research, University of Derby, Derby, United Kingdom; ^6^Awake to Wisdom Centre for Meditation and Mindfulness Research, Ragusa, Italy; ^7^Instituto de Investigaciones Sanitarias (IIS), Miguel Servet Hospital, Psychiatry Service, University of Zaragoza, Zaragoza, Spain

**Keywords:** attachment-based compassion therapy, fibromyalgia, randomized controlled trial, compassion, psychological flexibility

## Abstract

**Objective:**

There is a growing interest in evaluating the effectiveness of compassion interventions for treating psychological disorders. The present study evaluated the effectiveness of “attachment-based compassion therapy” (ABCT) in the treatment of fibromyalgia (FM), and the role of psychological flexibility as a mediator of improvements.

**Methods:**

A total of 42 patients with FM were randomly assigned to ABCT or relaxation (active control group). Both the intervention and control condition were combined with treatment as usual (TAU). The primary outcome was functional status (FIQ), and the secondary outcomes were clinical severity (CGI-S), pain catastrophizing (PCS), anxiety (HADS-A), depression (HADS-D), quality of life (EQ-5D), and psychological flexibility (AAQ-II). Differences between the groups were estimated using mixed-effects models, and mediation assessments were conducted using path analyses.

**Results:**

The ABCT group demonstrated superior outcomes compared to the relaxation group, including better FIQ values after treatment (*B* = −3.01; *p* = 0.003). Differences in FIQ were maintained at 3-month follow-up (*B* = −3.33; *p* = 0.001). The absolute risk reduction in ABCT compared to relaxation increased by 40.0%, with an NNT = 3 based on criteria of ≥50% FIQ reduction after treatment. Psychological flexibility had a significant mediating effect on improvements.

**Conclusion:**

These results suggest that ABCT combined with TAU appears to be effective in the treatment of FM symptoms.

**Clinical Trial Registration:**

http://ClinicalTrials.gov, identifier NCT02454244.

## Introduction

Fibromyalgia (FM) is a chronic and debilitating rheumatic condition, which includes symptoms of chronic pain, fatigue, and other physical and/or psychological issues such as disturbed sleep, cognitive problems, and distress (1). It is associated with mental health disorders such as anxiety and depression (2). The etiology of FM is complex and unknown and is still being explored. There are contradictory findings on determinants such as autonomic dysfunctions, imbalances in neurotransmitters and hormones, and external stressful events. The prevalence of FM has been estimated to be 2.9% in the European population (3), and its negative impact on work productivity and high usage of health-care resources has been highlighted (4).

While some studies have demonstrated the effectiveness of pharmacological treatments using antidepressants for improving pain and quality of life in FM patients (5), the maintenance of these benefits is questionable. Multicomponent interventions, including cognitive-behavioral therapy (CBT), seem to represent a more promising approach. CBT, which combines interventions from both cognitive and behavioral therapies, improves pain-coping capacity and reduces depressed mood and health care-seeking behaviors in FM patients (6, 7). It has been posited that (i) the cognitive components of CBT are likely to correspond to improvements in cognitive and affective domains, (ii) the behavioral components are likely to correspond to improvements in physical functioning and behavioral outcomes, and (iii) both cognitive and behavioral components assert a role in reducing pain intensity (8). However, the absence of clear, maintainable effects on the core symptoms of FM do not allow for a definitive statement regarding the effectiveness of this interventional approach (6, 9).

Acceptance and commitment therapy (ACT) reflects a “third wave” CBT approach that has been shown to improve pain, catastrophizing, acceptance, anxiety, depression, well-being, and quality of life in FM patients (10, 11). Furthermore, ACT has been shown to be cost-effective in comparison to pharmacological options, although it has not showed clear benefits in terms of the number of participants needed to treat (11). ACT is a transdiagnostic psychotherapeutic conglomerate that seems to lead to recovery from mental illness through the mediating role of psychological flexibility (i.e., the ability to function in the present moment without needless defense, or either persisting with or changing behavior in the pursuit of goals) (12). It includes a values-based activation component supported by the ongoing monitoring of present-moment experience and orientation toward acceptance, both of which are characteristics of mindfulness training (13).

In addition to being a component of ACT, mindfulness has also been delivered as a standalone intervention to patients with FM where it has been shown to lead to improve pain perception and quality of life (14, 15). In addition to increasing perceptual distance from distressing somatic and psychological stimuli, it has also been suggested that the spiritual aspects of mindfulness training can help to reduce core symptomatology (16). However, there is currently insufficient evidence supporting the use of mindfulness for patients with FM as a monotherapy alone (17).

Compassion is a psychological construct that involves (i) recognizing and understanding the universality of suffering, (ii) feeling emotionally connected with others’ suffering, (iii) tolerating uncomfortable feelings aroused as part of remaining open to and accepting of others’ suffering, and (iv) being motivated to act to alleviate suffering (18). Compassion has been a focus of research in recent years with findings indicating that it is associated with positive emotions (19) and can reduce stress-induced immune and behavioral responses (20). Several intervention protocols based on compassion have been described and used to treat psychological disorders such as anxiety and depression, with promising benefits (21). However, to date, such approaches have not been used to treat FM. A new protocol of compassion named “attachment-based compassion therapy” (ABCT) has recently been developed (22). It comprises eight sessions of 2.5 h duration, and includes exercises of mindfulness training and compassion such as receiving and offering compassion to (i) friends, (ii) individuals deemed to be problematic, (iii) unknown individuals, and (iv) oneself. ABCT is based on attachment theory (23), which provides a framework for understanding the links between close relationships and psychopathology. ABCT includes specific practices to identify how the parent–child relationship emerged in childhood. Where appropriate, it focusses on trying to solve possible maladjustments through reconciliation with parents.

### Aims of the Study

The aim of the present study was to examine the effectiveness of ABCT in the treatment of FM by exploiting synergies from the psychotherapeutic agents active in mindfulness, compassion, and the application of attachment theory. A low-intensity therapy based on relaxation (REL), which has been shown to improve FM symptoms but without clear clinical relevance (24), was deemed to be a suitable active control condition. A further aim was to assess the possible mediating role of psychological flexibility on clinical improvements in FM patients treated using the target intervention.

## Materials and Methods

### Design

A randomized controlled trial (RCT) with two treatment arms and a pre-, post-, and 3-month follow-up assessment was conducted. Adults suffering from FM were allocated to the REL or ABCT group using parallel assignment and a computer-generated randomization list. Findings from a third treatment arm outlined in the protocol (https://ClinicalTrials.gov Registration NCT02454244) that used mindfulness plus amygdala retraining are to be reported elsewhere.

### Participants

Fibromyalgia patients were recruited from primary health-care centres in Zaragoza, Spain. The inclusion criteria were: (1) male or female aged between 18 and 65 years, (2) able to read and understand Spanish, and (3) diagnosed with FM by a rheumatologist working for the Spanish National Health Service (SNHS). The exclusion criteria were: (1) aged <18 or >65 years, (2) presence of a severe Axis I psychiatric disorder (dementia, schizophrenia, paranoid disorder, alcohol, and/or drug use disorder) or severe somatic disorder that from the clinician’s point of view prevented the patient from effectively completing a psychological assessment, or (3) concurrent participation in another clinical trial. Antidepressant medication use was not considered to be an exclusion criterion so long as the participant agreed not to modify the pharmacological treatment that was prescribed during the study period (it was established that the treatment could be decreased but not increased).

Participants were recruited from January to March 2015. General practitioners (GPs) identified potential participants who were then interviewed at the same clinic by an independent researcher until the required sample size was achieved. This researcher assessed suitability according to the inclusion/exclusion criteria and provided potential participants with a general overview of the study. Participants were also informed of the confidentiality protocol and that they could withdraw from the study at any time without effecting the quality of the treatment administered by their GP. Witten informed consent was obtained from all study participants before assessment, which occurred from February to April 2015. The subsequent randomization occurred in April 2015 and the intervention was delivered from May to October 2015.

### Sample Size

We based our sample size estimation on an expected difference in the primary outcome [the Fibromyalgia Impact Questionnaire (FIQ)] of at least 20%, which represents a clinically relevant criterion. A previous descriptive study with the same context as the present work found an FIQ mean and SD of 70.8 and 15.2, respectively (25). Thus, a difference of 14.6 points across the groups was our target (0.95 SDs). Based on a 5% significance level and a statistical power of 80% in a two-tailed test, in order to detect this difference, we needed 18 participants in each group. We expected a maximum dropout rate of approximately 20%, so we increased the numbers to reach a total sample size of 42 participants.

### Interventions

#### Attachment-Based Compassion Therapy

This intervention included a compassion training program (22) that was adapted for FM patients. The intervention focused on augmenting the patient’s ability to be considerate and kind toward (i) themselves and their own experience (specifically their experience of suffering), and (ii) others’ experience of suffering. ABCT consists of 8 weekly 2-h sessions followed by three reminder monthly sessions. It involves practices of mindfulness and visualizations based on self-compassion and the attachment style that was generated in childhood (Table 1). The program includes daily homework assignments that take approximately 15–20 min to complete. The therapist in this group (MNG) was a psychologist who was specifically trained to conduct teacher training in ABCT.

**Table 1:** Session outlines for the treatment groups.

Sessions	Relaxation^a^		ABCT	
1	Visualizations I	Introduction to the different relaxation techniques and learning the distinct types and usefulness of each one of them. Initiation to guided relaxation through visualizations. Imagination of scenes in which one feels at peace and able to let go of stress and anxiety	Preparing compassion	Theoretical aspects of brain evolution, happiness, and suffering. Concept of compassion/self-compassion and elimination of mistaken beliefs. Participants are instructed in mindfulness practices such as breathing, and compassionate body scan. These practices help to regulate attention and emphasize compassionate aspects within oneself. They are a core element of the program
2	Visualizations II	Imagination training. Deepening in guided relaxation through visualizations. Knowing the effects that this type of relaxation has in the body and mind. Learning in which situations it might be useful. Using landscape images to achieve states of relaxation	Self-esteem and compassion	Mindfulness and compassion. Differences with self-esteem. How to manage and cope with fear of compassion. Practices to try to connect with affection and compassion with other beings, and to try to generate feelings of security toward oneself. It is analyzed whether participants have previously developed a mental referent figure in their life, to resort to in distressful situations
3	Visualizations III	Working with emotions through imagination. One visualizes flying in a balloon. Emotional burdens are difficult to alleviate, so they are symbolically released and thrown down, helping the mind to be free of emotions that cause discomfort	Developing my compassionate world	Action mechanism of compassion. Importance of replacing self-criticism with self-compassion. Development of a core element of compassion such as the figure of secure attachment. Replacing the critical voice with a more compassionate and tolerant one. Importance of acceptance in life
4	Autogenic relaxation I	Autogenic relaxation initiation. Knowing the effects this type of relaxation has in the body and mind. Imagining a ball of light and heat to aid sensations of relaxation	Relationships and compassion	Parenting models during childhood. Understanding that relationships with parents generate different ways of relating to the world. Awareness of the emotional bond developed toward parents during childhood, as well as their implications for the emotional functioning of adulthood, and the capacity we have to be able to receive affection from others
5	Autogenic relaxation II	Deepening in autogenic relaxation. Learning in which situations it could be useful and how to use it. Working on heaviness sensations. Body sweeping, especially emphasizing feelings of heaviness and relaxation	Working on ourselves	Reconstruction of a secure attachment model, modifying our relationships with ourselves and with others by compassion. Practices to become aware of our own ability to give affection to others and ourselves. Reconciliation with parents (where appropriate)
6	Progressive relaxation	Initiation to the progressive relaxation. Explanation of this technique and the different benefits of it, such as learning to locate and address bodily tension. Tensing and relaxing the muscles to become aware of the different sensations in both states (i.e., tense vs. relaxed)	Advanced compassion I	Forgiveness and common barriers to compassion. Importance of forgiveness toward oneself and others. Forgiveness through meditation: (1) asking forgiveness of others, (2) forgiving oneself, (3) forgiving others for wrongs received. Values guide activation to reduce suffering
7	Breathing I	Learning to use breathing exercises. Knowing the benefits that this type of relaxation has in the body and mind. Deep inspiration and exhalation. Using breathing to calm anxiety	Advanced compassion II	Envy and the importance of developing an attachment figure based on oneself. How to manage difficult relationships. Trying to understand other’s suffering in order to develop applied compassion in daily life
*8*	Breathing II	Deepening in breathing exercises and their benefits. Learning different techniques and exercises based on pulmonary or external deep breathing, and on internal or cellular respiration	Transmitting compassion toward others	Equanimity, a quality fruit of compassion practice. How to maintain compassion exercises throughout life. Practices to develop equanimity: we are all the same, the fallacy of categories, giving gratitude. Maintenance of compassion toward others and ourselves

*^a^Relaxation group*.

*ABCT, attachment-based compassion therapy*.

#### Relaxation Group (REL)

This arm constituted the active control condition. Participants received a low-intensity and non-specific intervention based on 8 weekly 2-h sessions involving several grouped relaxation techniques such as imagery, autogenic training, progressive muscle relaxation, and breathing (Table 1). The program includes daily homework assignments that take approximately 15–20 min to complete. After completing the 8 weekly sessions, participants receive a further three monthly sessions as reminders. For ethical reasons, following completion of the study, participants in this group had the option to receive ABCT. The therapist (Marta Puebla-Guedea) was a psychologist trained in relaxation techniques.

### Procedure

A researcher who had no other involvement in the study generated the random allocation sequence to determine group assignment. Randomization was implemented *via* telephone, and the allocation details were concealed from the other researchers until all participants had been assigned. Participants agreed to participate prior to randomization and were not informed of group allocation until after completion of baseline assessments. Likewise, participants were not informed of which allocation condition was the target intervention. The outcome assessor remained blind as to participant allocation. The RCT was conducted according to the “Initiative on Methods, Measurement and Pain Assessment in Clinical Trials” (IMMPACT) recommendations and the “Consolidated Standards of Reporting Trials” (CONSORT) guidelines. The study followed the Helsinki Convention norms and subsequent modifications, Declaration of Madrid of the World Psychiatric Association and Uniform Requirements for Manuscripts Submitted to Bio-Medical Journals. The study protocol was approved by the ethical review board of the regional health authority of Aragon, Spain (PI15/0049; 01/04/2015). The study was conducted from January 2015 (start of recruitment) to January 2016 (end-of-study assessment).

Each of the study arms were combined with treatment as usual (TAU) provided by the SNHS for FM patients. This treatment is offered by the corresponding GP and consists of administering pregabalin or other drugs for pain as well as antidepressants such as duloxetine (or another similar SNRI) if there is a diagnosis of depression. TAU can also include pharmacological treatments for insomnia and fatigue. Furthermore, GPs may refer the patient to a rheumatologist, psychiatrist, and/or other specialist as required. Psychological treatment is not provided by the SNHS and pharmacotherapy is the frontline option (although it is occasionally offered in conjunction with non-pharmacological rehabilitative techniques).

### Measurements

Participants completed a sociodemographic and clinical survey based on a paper-and-pencil battery of questionnaires that were assessed at baseline, posttreatment (8 weeks after baseline), and 3-month follow-up. The following sociodemographic information was collected: gender, age, marital status (with a partner vs. not with a partner), dwelling (own home vs. rent home), education level (primary, secondary, university), and employment status (unemployed, employed, sick leave/inability).

The Fibromyalgia Impact Questionnaire (FIQ), which has been proposed as a primary efficacy end-point measure in FM clinical trials, was the primary outcome. It is a self-report questionnaire that comprises 10 items to measure the health status of patients with FM. The first item focusses on patients’ ability to perform physical activities. The next two items require patients to indicate the number of days in the past week that they felt good as well as how many days of work they missed. The remaining seven items are measured using the Visual Analog Scale (VAS) and concern ability to work, pain, fatigue, morning tiredness, stiffness, anxiety, and depression. The total score is subjected to normalization and ranges from 0 to 100, with higher scores indicating greater functional impairment. The current study used a Spanish-language-validated version of the FIQ that demonstrates good psychometric properties (26).

Based on previous studies (27), we chose the clinical global impression as a proxy for the general well-being of FM patients, and we selected a number of secondary measures likely to be sensitive to the effects of the intervention. These comprised pain catastrophizing, anxiety and depressive symptoms, quality of life, and psychological flexibility, the latter of which appears to function as a transdiagnostic mediating factor for mental health and medical conditions (10, 28).

The Clinical Global Impression-Severity Scale (CGI-S) is a 7-point scale that requires the clinician to rate the severity of the patient’s illness at the time of assessment relative to the clinician’s past experience with patients that have the same diagnosis. Based on their clinical experience, the mental illness of a patient is assessed in terms of severity as: normal, not at all ill (1); borderline, mentally ill (2); mildly ill (3); moderately ill (4); markedly ill (5); severely ill (6); or extremely ill (7). It is one of the most widely used brief assessment tools in psychiatry, and it has been used in previous studies of FM (27).

The Pain Catastrophizing Scale (PCS) is a self-report questionnaire that measures pain catastrophizing (one of the main symptoms in FM) and comprises 13 items relating to focusing excessively on pain sensations, magnifying the threat value of pain sensations, and perceiving oneself as unable to control the intensity of pain. Items are rated in relation to their frequency of occurrence on a 5-point scale, from never (0) to almost always (4). The PCS total score is computed as the algebraic sum of the ratings for each item, and varies from 0 to 52. Higher scores reflect a greater degree of pain catastrophizing. The Spanish version of the PCS, which has demonstrated good psychometric properties in FM patients, was used in the present study (29).

The Hospital Anxiety and Depression Scale (HADS) is a self-report questionnaire that quantifies the severity of anxiety and depressive symptoms in community and hospital settings. It includes 14 items that are rated on a 4-point Likert-type scale and has two independent subscales (HADS-A and HADS-D). Each subscale ranges from 0 to 21. Higher scores indicate more severe symptoms. This scale is considered to be one of the best questionnaires for assessing anxiety and depressive symptoms in patients with pain disorders. The Spanish-validated version of the HADS, which has good psychometric properties when used with Spanish FM patients, was administered in the present study (30).

The VAS of the EuroQol (EQ-5D) is a vertical line that is similar to a thermometer on which the best and worst possible health states are scored 100 or 0, respectively. It is a standardized, non-disease-specific instrument for describing and valuing health-related status in terms of quality of life. The Spanish version of the EQ-5D is a valid and practical outcome measure in clinical research and can discriminate between healthy individuals and chronic patients (31).

The Acceptance and Action Questionnaire (AAQ-II) is a self-report tool that evaluates psychological flexibility as the (i) unwillingness to experience unwanted emotions and thoughts and (ii) inability to be in the present moment or use values-directed actions when experiencing distressing psychological events. It consists of seven items and responses are provided using a 7-point Likert scale (from 1 = never to 7 = always). The final score ranges from 7 to 49, with higher scores indicative of lower levels of psychological flexibility. The Spanish version of the AAQ-II, which has emerged as a reliable and valid measure of psychological flexibility, was used in the present study (32).

### Statistical Analyses

First, we used means and SD, medians, and interquartile range, and frequencies and percentages to assess the balance of sociodemographic and clinical outcomes across study arms at baseline. For pretreatment comparisons, one-way analyses of variance for continuous variables, Mann–Whitney *U* for ordinal data, and the Fisher exact probability test for categorical variables were used.

The primary between-group analysis to assess the treatment effect was performed on an intention-to-treat basis with FIQ total score as a continuous variable. It involved using linear mixed-effects models in which restricted maximum likelihood regression (REML) was used to account for the correlation between repeated measures for each individual. REML produces less biased estimates of variance parameters when using small sample sizes or unbalanced data (33). Regression coefficients (*B*) and 95% confidence intervals (95% CI) were calculated for the Group × Time interaction between groups at posttest and 3-month follow-up. We reported the effect size (ES) for each pairwise comparison, using the pooled pretest SD to weight the differences in the pre-post means, and to correct for the population estimate (34). The rule of thumb is usually that an ES of 0.20 is small, 0.50 is medium, and 0.80 is large. Separate models were estimated for each of the secondary outcomes using the same analytical strategy. Only the total score of each scale was taken into account to keep the statistical analyses as parsimonious as possible. Sensitivity analyses were conducted to assess the effects of missing data and to examine how robust our primary and secondary analyses were. After ensuring that the data were missing at random, missing values were replaced using the baseline observation carried forward (BOCF) approach as well as multiple imputations based on chained equations.

We differentiated participants into two categories (responders/non-responders to treatment) using two different cut-off criteria: (a) ≥20% reduction in the pre–post FIQ total score and (b) ≥50% reduction in the pre-post FIQ total score (35, 36). These two classifications were used to calculate the number needed to treat (NNT) in the ABCT group compared to the Relaxation group. NNT refers to the estimated number of patients who need to be treated with the new proposed treatment (i.e., rather than the control comparison treatment) for one additional patient to benefit. A confidence interval (95% CI) for each NNT was calculated. This index allows findings from RCTs to be more meaningful to clinicians.

We examined whether the effect of ABCT on primary and secondary outcomes at 3-month follow-up was mediated through changes in psychological flexibility at posttest. We calculated pre–post change scores on the AAQ-II and pre-follow-up change scores on the rest of the outcomes. Bivariate Pearson correlations between changes in AAQ-II and the other outcomes were estimated. We explored the direct and indirect relationships between the treatment condition (independent variable), AAQ-II (mediator), and clinical outcomes (dependent variables) using path analysis. The direct paths between the treatment condition and clinical outcomes and the indirect effect path through AAQ-II were tested in all the path analysis models. Participants with missing data were excluded. Regression coefficients (B) of bias-corrected bootstrapped indirect effects were calculated as well as their SEs and 95% CIs. This procedure produces a test that can be applied to small samples, overcoming possible problems of asymmetry in the distribution of indirect effects (37). Parameters of indirect effects were considered statistically significant when the 95% CI did not include 0.

A 5% significance level was used in all two-tailed tests and Bonferroni’s multiple comparisons criterion (for the primary but not secondary outcomes) were applied to balance type I and type II errors. All of the analyses were performed using SPSS-19 and Stata-12.

## Results

### Participants’ Flow and Compliance

**Figure 1:**
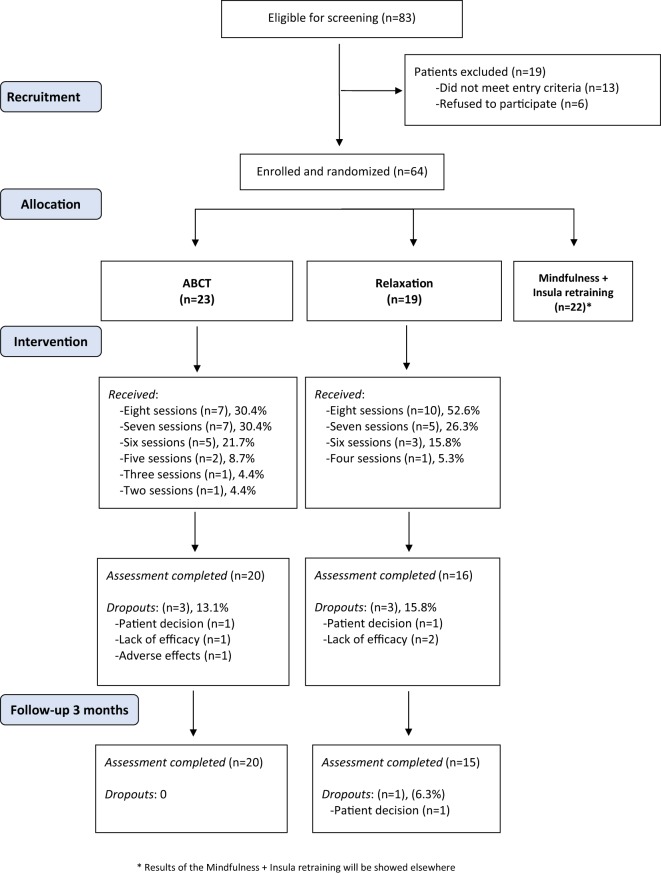
Flowchart of participants in the randomized controlled trial.

Of the 83 patients who were eligible for screening, 19 patients were excluded (Figure 1). The main reasons for exclusion were (i) not diagnosed by a rheumatologist (*n* = 8), (ii) older than 65 years (*n* = 2), and (iii) suffering from a psychiatric disorder (schizophrenia; *n* = 1) or severe medical disorder (lupus; *n* = 2). Six patients refused to participate. Of the 64 participants who were eligible and randomly allocated to a group, 23 were assigned to ABCT and 19 to Relaxation (22 participants were assigned to a mindfulness + amygdala group, whose results will be reported elsewhere). The mean percentage of ABCT sessions attended was 81.5% (SD = 19.90), whereas 90.1% (SD = 13.55) of the Relaxation sessions were attended [with no significant differences between groups *t*(40) = 1.60, *p* = 0.117]. The level of homework accomplishment was similar among groups. Specifically, 17 participants (85.0%) in the ABCT group and 13 (81.3%) in the Relaxation (Fisher’s test, *p* = 0.742) group completed their homework. The majority of participants were taking medication during the treatment period [ABCT = 20 (100%); Relaxation = 15 (93.8%); Fisher’s test, *p* = 0.444]. The completion rate was high with 36 (85.7%) and 35 (83.3%) participants completing the posttreatment and the 3-month follow-up assessments, respectively. Seven participants dropped out of the study (3 in the ABCT group, and 4 in the Relaxation group). Therefore, a total of 20 (87.0%) participants in the ABCT, and 15 (78.9%) in the Relaxation group completed the study (Fisher’s test, *p* = 0.682). Only relationship status was significantly associated with attrition [missing with a partner = 4 (9.3%), missing without a partner = 7 (33.3%); Fisher’s test, *p* = 0.031]. Given that no baseline-level differences in other sociodemographic or primary/secondary outcomes were observed between completers and non-completers, dropouts were considered to be at random.

### Group Baseline Characteristics

There were no significant differences between the groups on any sociodemographic or clinical variable at baseline (Table 2). All participants were women in their early fifties, living in their own home, and with a secondary school level of education. Participants were mostly unemployed and in a relationship. The average rating was severe on FM impact (FIQ) and markedly ill for the clinical global impression (CGI-S). Participants’ anxiety and depression levels (HADS) were high and their perceived health status (EQ-5D) was relatively fair.

**Table 2:** Baseline sociodemographic and clinical features of patients by group.^a^

Characteristics at baseline	REL (*n* = 19)	ABCT (*n* = 23)	Test statistic	*p*
Sex, female	19 (100)	23 (100)	(^b^)	1.000
Age	52.21 (5.95)	50.83 (8.70)	*t* = 0.56 (40)	0.559
Relationships, with partner	13 (68.4)	18 (78.3)	(^b^)	0.504
Dwelling, own home	17 (89.5)	21 (91.3)	(^b^)	1.000
**Education**				
Primary	4 (21.1)	10 (43.5)	(^b^)	0.312
Secondary	8 (42.1)	8 (34.8)		
University	7 (36.8)	5 (21.7)		
**Employment**				
Housework	6 (31.6)	10 (43.5)	(^b^)	0.543
Employed	3 (15.8)	4 (17.4)		
Sick leave/inability	5 (26.3)	7 (30.4)		
Unemployed	5 (26.3)	2 (8.7)		
**Fibromyalgia impact**				
FIQ (0–100)	62.83 (18.41)	68.49 (16.26)	*t* = 1.06 (40)	0.297
**Clinical severity**				
CGI-S (1−7)	4.32 (1.16)	4.52 (0.85)	*t* = 0.67 (40)	0.509
**Pain catastrophizing**				
PCS (0−52)	25.00 (10.92)	24.04 (12.10)	*t* = 0.27 (40)	0.791
**Anxiety and depression**				
HADS-A (0−21)	11.37 (5.40)	13.65 (4.06)	*t* = 1.56 (40)	0.126
HADS-D (0−21)	8.05 (6.03)	10.09 (3.19)	*t* = 1.33 (26)^c^	0.197
**Perceived health**				
EQ-5D (0−100)	54.00 (20.19)	47.83 (12.78)	*t* = 1.16 (29)^c^	0.258
**Psychological flexibility**				
AAQ-II (10−70)	37.32 (13.34)	41.78 (10.33)	*t* = 1.22 (40)	0.229

*REL, relaxation; ABCT, attachment-based compassion therapy; FIQ, fibromyalgia impact questionnaire; CGI-S, Clinical Global Impression Severity; PCS, pain catastrophizing scale; HADS-A, hospital anxiety and depression scale-anxiety; HADS-D, hospital anxiety and depression scale-depression; EQ-5D, visual analog scale of EuroQol; AAQ-II, acceptance and action questionnaire*.

*^a^Data are presented as means (SD) or *n* (%); *p, p*-value associated with the comparison*.

*^b^Fisher probability test*.

*^c^No equal variances have been assumed*.

### Patient Preferences, Credibility of Therapies, and Quality of Interventions

The preferred choice of intervention of each participant was evaluated before group allocation. A similar distribution was observed among the study arms. More specifically, 8 (42.1%) participants in the Relaxation group, and 13 (56.5%) in the ABCT condition, had no specific preference (Fisher’s test, *p* = 0.536). The credibility of the therapy was evaluated from 0 (minimum credibility) to 10 (maximum credibility). Each participant assessed the intervention after receiving it, and no significant differences were found among groups [Relaxation Median = 8 (Q1 = 7/Q3 = 9); ABCT Median = 8 (Q1 = 8/Q3 = 8); *Z* = 0.71; *p* = 0.476]. All sessions were audio recorded in every intervention group, and one of the researchers, who was a senior therapist (Javier García-Campayo), randomly attended and assessed two sessions of each study arm to confirm that the psychological treatments followed the corresponding protocol with a high level of fidelity.

### Efficacy in the Primary and Secondary Outcomes

Table 3 shows the descriptive statistics and between-group analyses. There were significant differences in FIQ, with ABCT performing better than Relaxation at posttest (*B* = −22.07, *Z* = −3.01, *p* = 0.003) and at 3-month follow-up (*B* = −24.78, *Z* = −3.33, *p* = 0.001), with high ESs (*d* = 1.33 and *d* = 1.38, respectively). Results involving the secondary outcomes showed a similar pattern of differences, except for PCS, which showed no significant differences at any time. Models with imputed missing values using the BOCF method showed small reductions in regression coefficients, but results remained unaltered. The chained equations method reinforced significant differences in FIQ, HADS-D (at posttest), EQ-5D, and AAQ-II (Table S1 in Supplementary Material).

**Table 3:** Descriptive statistics and between-group analyses with observed data.

Outcomes/time points	REL (*n* = 15) Mn (SD)	ABCT (*n* = 20) Mn (SD)	*d*	*B* (95% CI)	*Z*	*p*
**FIQ**						
Baseline	61.12 (20.21)	68.37 (17.38)				
Posttreatment	61.22 (25.90)	43.51 (10.31)	1.33	−22.07 (−36.46 to −7.48)	−3.01	0.003
Follow-up	67.82 (17.77)	49.13 (15.07)	1.38	−24.78 (−39.35 to −10.20)	−3.33	0.001
**CGI-S**						
Baseline	4.27 (1.28)	4.60 (0.82)				
Posttreatment	4.33 (0.82)	3.65 (0.49)	0.96	−1.11 (−1.85 to −0.36)	−2.91	0.004
Follow-up	4.07 (0.80)	3.20 (0.70)	1.14	−0.91 (−1.67 to −0.15)	−2.35	0.019
**PCS**						
Baseline	25.93 (10.14)	23.75 (12.79)				
Posttreatment	23.47 (14.49)	18.55 (11.94)	0.23	0.17 (−5.93 to 6.26)	0.05	0.957
Follow-up	23.53 (13.58)	18.05 (10.50)	0.28	−1.89 (−8.61 to 4.84)	−0.55	0.582
**HADS-A**						
Baseline	11.53 (6.06)	13.95 (4.27)				
Posttreatment	10.53 (5.24)	7.65 (2.62)	1.03	−4.62 (−7.12 to −2.12)	−3.63	<0.001
Follow-up	9.80 (4.84)	7.60 (2.82)	0.90	−3.08 (−5.63 to −0.53)	−2.37	0.017
**HADS-D**						
Baseline	8.33 (6.67)	10.35 (3.28)				
Posttreatment	7.53 (4.81)	4.80 (2.84)	0.94	−4.41 (−6.82 to −2.00)	−3.59	<0.001
Follow-up	7.80 (5.99)	4.70 (2.00)	1.01	−3.82 (−6.28 to −1.36)	−3.04	0.002
**EQ-5D**						
Baseline	53.07 (21.71)	48.25 (13.01)				
Posttreatment	56.87 (18.95)	66.65 (10.77)	−0.84	18.01 (6.56–29.47)	3.08	0.002
Follow-up	61.67 (15.66)	71.65 (7.34)	−0.85	14.83 (3.13–26.53)	2.49	0.013
**AAQ-II**						
Baseline	38.00 (14.23)	42.35 (10.54)				
Posttreatment	39.07 (13.90)	29.50 (6.94)	1.13	−13.04 (−20.53 to −5.55)	−3.41	0.001
Follow-up	37.00 (12.78)	26.80 (5.82)	1.18	−14.90 (−22.49 to −7.31)	−3.85	<0.001

*REL, relaxation; ABCT, attachment-based compassion therapy; Mn, mean; d, Cohen’s d effect size corrected for repeated measures; B, regression coefficient; 95% CI, 95% confidence interval; Z, value of the Z statistic; p, p-value for each comparison; FIQ, Fibromyalgia Impact Questionnaire; CGI-S, Clinical Global Impression Severity; PCS, pain catastrophizing scale; HADS-A, Hospital Anxiety and Depression Scale-Anxiety; HADS-D, Hospital Anxiety and Depression Scale-Depression; EQ-5D, Visual Analog Scale of EuroQol; AAQ-II, Acceptance and Action Questionnaire*.

### Absolute Risk Reduction and Number Needed to Treat

A total of 75.0% of participants in the ABCT group and 18.8% of participants in the Relaxation group [15 of 20 (ABCT) and 3 of 16 (Relaxation) who completed pre- and post-assessments] reached the criterion of ≥20% FIQ reduction after treatment. Therefore, the absolute risk reduction in ABCT compared to Relaxation increased by 56.3% (95% CI = 29.3–83.2%), with an NNT = 2 (95% CI = 1.2–3.4%). Furthermore, 40.0 and 0.0% of the participants in the ABCT and Relaxation groups (8 of 20, and 0 of 16, who completed pre- and post-assessments), respectively, reached the criterion of ≥50% FIQ reduction after treatment. Therefore, the absolute risk reduction in ABCT compared to Relaxation increased by 40.0% (95% CI = 18.5–61.5%), with an NNT = 3 (95% CI = 1.6–5.4%).

### Indirect Effects of Psychological Flexibility

There was a significant change from baseline to posttreatment in AAQ-II in the ABCT [*t*(19) = 4.75, *p* < 0.001] but not in the Relaxation [*t*(15) = −0.57, *p* = 0.580] condition. Considering the total sample, correlations between pre-post changes in AAQ-II and pre-follow-up changes in clinical outcomes were significant for the FIQ (*r* = 0.46, *p* < 0.001), HADS-A (*r* = 0.51, *p* < 0.001), HADS-D (*r* = 0.54, *p* < 0.001), and EQ-5D (*r* = −0.29, *p* = 0.037), but they were not significant for the CGI-S (*r* = 0.23, *p* = 0.094), and PCS (*r* = 0.04, *p* = 0.778). With the exception of PCS, there were significant direct paths between the study condition and clinical outcomes. The study condition predicted the change in AAQ-II (*B* = −13.92, *p* = 0.001), which in turn predicted the change in FIQ (*B* = 1.15, *p* = 0.001), HADS-A (*B* = 0.13, *p* = 0.020), and HADS-D (*B* = 0.20, *p* < 0.001). The percentage of the study condition effect on FIQ mediated through AAQ-II was 62.0% [the non-significant direct path (*B* = 8.19; *p* = 0.232) between the study condition and FIQ accounted for the remaining 38.0%], on HADS-A it was 38.1% [the significant direct path (*B* = −2.86; *p* = 0.044) between the study condition and HADS-A accounted for the remaining 61.9%], and on HADS-D it was 54.5% [the significant direct path (*B* = −2.33; *p* = 0.045) between the study condition and HADS-D accounted for the remaining 45.5%]. These values were not compatible with the idea of full mediation. There were not significant direct effects between study condition and PCS (−3.30; *p* = 0.274), but there were significant indirect effects through AAQ-II (Table 4).

**Table 4:** Indirect effects of AAQ-II (*n* = 35).

Outcomes	*B*	SE	95% CI	
FIQ	−15.98	6.05	−29.52	−5.91
CGI-S	−0.13	0.22	−0.56	0.32
PCS	−2.76	1.59	−6.72	−0.30
HADS-A	−1.76	1.01	−4.14	−0.10
HADS-D	−2.79	0.85	−4.82	−1.41
EQ-5D	3.94	4.17	−2.72	14.25

*B, regression coefficient of the bootstrapped indirect effect; 95% CI, bias corrected confidence interval of indirect effects; FIQ, Fibromyalgia Impact Questionnaire; CGI-S, Clinical Global Impression Severity; PCS, Pain Catastrophizing Scale; HADS-A, Hospital Anxiety and Depression Scale-Anxiety; HADS-D, Hospital Anxiety and Depression Scale-Depression; EQ-5D, Visual Analog Scale of EuroQol*.

## Discussion

This is the first study to evaluate the efficacy of ABCT for treating FM. ABCT can be considered a “third-generation” protocol because it seeks to foster interpersonal virtues, such as compassion, forgiveness, and gratitude (38). The program is specifically based on attachment styles, a psychoanalytical concept that describes the relationship children develop with their parents and that influences the interpersonal relationships and self-image they subsequently develop. Through the awareness and clarification of values in the fields of family, friendship, love, work, spirituality, and general well-being, ABCT encourages a reorientation of the person’s life by the acceptance of his/her specific situation, improving activation and functioning in each specific context (22).

Attachment-based compassion therapy plus TAU produced greater increases in the general health status of FM patients (i.e., as measured by the FIQ) compared to a group of relaxation practices added to TAU. These improvements were maintained in a significant way at 3-month follow-up. Additional significant improvements in the ABCT group were also observed in clinical severity, anxiety, depression, quality of life, and psychological flexibility. Surprisingly, they were not observed in pain catastrophizing, a keystone variable of this syndrome, which has been reduced in FM patients by more cognitively complex approaches such as ACT (10). In general, the ESs obtained in the present study were larger than those achieved when treating FM using CBT (39) or mindfulness alone (15). For general health status and quality of life, ESs were similar to those observed in a previous study using ACT, but they were higher for anxiety and depression and lower for pain catastrophizing (10). The clinical value of ABCT in terms of the NNT was apparent, and varied from 2 to 3 depending on the cut-off point considered. Therefore, ABCT might be considered as an effective treatment for reducing FM symptoms (40).

Mediation analyses showed that pre–post changes in psychological flexibility partially mediated the relationships between study condition and FM global health status, pain catastrophizing, anxiety, and depression at follow-up. These results are in line with those obtained in other studies examining psychological flexibility as a transdiagnostic key factor of psychopathological change processes in complex and chronic conditions (41). Overcoming the dominance of private experiences over chosen values and contingencies in guiding action might be a core element underlying psychotherapeutic change (42), and it could be related to the values-based activation, acceptance, and persistence that are characteristics of third wave therapies such as ACT (10, 12). However, our results suggest that ABCT therapeutic processes might also depend on other mediators, given that psychological flexibility did not explain all of the improvements. In this sense, future research could seek to evaluate the possible mediating role of other variables related to the main ingredients of ABCT, such as attachment, compassion, and mindfulness facets. Notwithstanding this, it is not possible to discard other mechanisms of action operating through psychological flexibility, and it would be worth investigating in future research, for instance, the extent of the synergistic interplay of this variable (43).

Despite the aforementioned treatment effects, findings should be interpreted in light of their limitations. First, although the final sample size used was within the limits of our power calculation, it was relatively small when considering drop out. Although the target was to detect outcome differences with a clear clinical meaning, this could lead to findings of no significant differences with moderate ESs in some cases. Second, the study did not control for the possible influences of the therapist and thus it remains unknown how therapist variables could have affected the results. This limitation could be addressed in future studies by estimating the percentage of variance explained by the therapists’ influence. Finally, the study did not compare the intervention with other psychological treatments that have demonstrated effectiveness for FM such as CBT or ACT (9, 10). In this sense, future studies could compare the effectiveness and cost-effectiveness of the proposed intervention with other active control conditions that are similar in terms of duration, allegiance, and expectations, etc. It would be also interesting to investigate and separate the active ABCT ingredients (among which might be attachment, compassion, or mindfulness) in order to investigate how they affect different subgroups of FM patients (44, 45).

In terms of the study strengths, it is worth highlighting that participants’ preferences, credibility of the therapies, and quality of the interventions were of a similar distribution between groups. Therefore, it was possible to circumvent some of the aforementioned limitations without statistical control procedures, which usually involve a loss of power when attempting to detect differences using small sample sizes. Moreover, the use of two different methods to impute dropouts and the triangulation of results using both observational and imputing data procedures reinforced the strength of our findings.

The ABCT third-generation intervention, added to TAU, proved to be more effective than TAU plus relaxation at posttest and 3-month follow-up in the treatment of FM patients. Psychological flexibility appears to be a mediating treatment factor although it remains unclear whether other variables—including those related to attachment, compassion, and mindfulness—might also exert a mediating role in improving FM symptomatology. Findings suggest that studies investigating the cost-effectiveness of ABCT for treating FM are warranted.

## Ethics Statement

Participants agreed to participate prior to randomization and were not informed of group allocation until after completion of baseline assessments. Likewise, participants were not informed of which allocation condition was the target intervention. The outcome assessor remained blind as to participant allocation. The RCT was conducted according to the “Initiative on Methods, Measurement, and Pain Assessment in Clinical Trials” (IMMPACT) recommendations and the “Consolidated Standards of Reporting Trials” (CONSORT) guidelines. The study followed the Helsinki Convention norms and subsequent modifications, Declaration of Madrid of the World Psychiatric Association and Uniform Requirements for Manuscripts Submitted to Bio-Medical Journals. The study protocol was approved by the ethical review board of the regional health authority of Aragon, Spain (PI15/0049; 01/04/2015). The study was conducted from January 2015 (start of recruitment) to January 2016 (end-of-study assessment).

## Author Contributions

JG-C developed the study concept. JM-M, MN-G, MP-G, and JG-C contributed to the study design. MN-G and MP-G implemented the interventions. JM-M performed the data analysis and interpretation of results under the supervision of JG-C and drafted the manuscript. WG, ES, and JL provided critical revisions. All authors approved the final version of the manuscript for submission.

## Conflict of Interest Statement

The authors declare that the research was conducted in the absence of any commercial or financial relationships that could be construed as a potential conflict of interest.

## Supplementary Material

Analyses with imputed data using both the BOCF method and chained equations.
