# Supplementary material for: Efficacy of “Attachment-Based Compassion Therapy” in the Treatment of Fibromyalgia: A Randomized Controlled Trial
Source: Front Psychiatry. 2018 Jan 16;8:307. doi: 10.3389/fpsyt.2017.00307 (PMC5775966; doi:10.3389/fpsyt.2017.00307)
Supplement: Supplementary file 1 [file table_1.docx]

**Supplemental Material**

**Descriptive statistics and between-group analyses with imputed data using the BOCF method**

|  | **REL (n=19)**  Mn (SD) | **ABCT (n=23)**  Mn (SD) | **d** | **B (95% CI)** | **z** | ***p*** |
| --- | --- | --- | --- | --- | --- | --- |
| *FIQ* |  |  |  |  |  |  |
| Post-Treatment | 63.30 (23.39) | 46.87 (13.18) | 1.27 | -22.09 (-35.10 – -9.09) | -3.33 | 0.001 |
| Follow-up | 68.52 (15.99) | 51.76 (15.74) | 1.29 | -22.42 (-35.43 – -9.42) | -3.38 | 0.001 |
| *CGI-S* |  |  |  |  |  |  |
| Post-Treatment | 4.32 (0.82) | 3.70 (0.56) | 0.81 | -0.83 (-1.50 – -0.15) | -2.41 | 0.016 |
| Follow-up | 4.16 (0.77) | 3.30 (0.77) | 1.05 | -1.06 (-1.73 – -0.39) | -3.09 | 0.002 |
| *PCS* |  |  |  |  |  |  |
| Post-Treatment | 21.90 (14.69) | 19.52 (11.58) | 0.12 | -1.42 (-6.72 – 3.89) | -0.52 | 0.601 |
| Follow-up | 23.11 (13.41) | 19.09 (10.35) | 0.26 | -3.06 (-8.37 – 2.24) | -1.13 | 0.258 |
| *HADS-A* |  |  |  |  |  |  |
| Post-Treatment | 10.37 (4.79) | 8.17 (2.82) | 0.95 | -4.48 (-8.80 – -2.16) | -3.78 | <0.001 |
| Follow-up | 9.79 (4.44) | 8.13 (2.99) | 0.83 | -3.94 (-6.26 – -1.62) | -3.33 | 0.001 |
| *HADS-D* |  |  |  |  |  |  |
| Post-Treatment | 7.79 (4.40) | 5.26 (2.97) | 0.97 | -4.56 (-6.72 – -2.40) | -4.14 | <0.001 |
| Follow-up | 7.63 (5.43) | 5.17 (2.33) | 0.95 | -4.49 (-6.65 – -2.33) | -4.07 | <0.001 |
| *EQ-5D* |  |  |  |  |  |  |
| Post-Treatment | 57.53 (18.35) | 63.83 (13.10) | -0.75 | 12.43 (1.94 – 23.01) | 2.32 | 0.020 |
| Follow-up | 60.79 (15.21) | 68.17 (12.11) | -0.81 | 13.56 (3.02 – 24.09) | 2.52 | 0.012 |
| *AAQ-II* |  |  |  |  |  |  |
| Post-Treatment | 38.53 (12.95) | 30.61 (7.66) | 1.04 | -12.38 (-19.17 – -5.60) | -3.58 | <0.001 |
| Follow-up | 36.53 (12.10) | 28.26 (7.25) | 1.07 | -12.73 (-19.52 – -5.94) | -3.68 | <0.001 |

REL: Relaxation; ABCT: Attachment-Based Compassion Therapy; Mn: mean; SD: standard deviation; d: Cohen’s d effect size corrected for repeated measures; B: regression coefficient; 95% CI: 95% confidence interval; Z: value of the Z statistic; p: p-value for each comparison; FIQ: Fibromyalgia Impact Questionnaire; CGI-S: Clinical Global Impression Severity; PCS: Pain Catastrophizing Scale; HADS-A: Hospital Anxiety and Depression Scale-Anxiety; HADS-D: Hospital Anxiety and Depression Scale-Depression; EQ-5D: Visual Analogue Scale of EuroQol; AAQ-II: Acceptance and Action Questionnaire.

**Descriptive statistics and between-group analyses with imputed data using chained equations**

|  | **REL (n=19)**  Mn (SD) | **ABCT (n=23)**  Mn (SD) | **d** | **B (95% CI)** | **z** | ***p*** |
| --- | --- | --- | --- | --- | --- | --- |
| *FIQ* |  |  |  |  |  |  |
| Post-Treatment | 54.95 (30.73) | 38.88 (36.76) | 1.25 | -16.99 (-27.59 – -6.38) | -3.14 | 0.002 |
| Follow-up | 66.72 (40.48) | 54.16 (36.40) | 1.05 | -13.59 (-24.20 – -2.97) | -2.51 | 0.012 |
| *CGI-S* |  |  |  |  |  |  |
| Post-Treatment | 3.74 (1.37) | 3.75 (1.26) | 0.19 | 0.33 (-0.15 – 0.80) | 1.34 | 0.181 |
| Follow-up | 3.77 (1.75) | 3.16 (1.84) | 0.80 | -0.29 (-0.77 – 0.19) | -1.19 | 0.234 |
| *PCS* |  |  |  |  |  |  |
| Post-Treatment | 11.92 (18.18) | 15.42 (16.30) | -0.38 | 0.24 (-5.89 – 6.36) | 0.08 | 0.940 |
| Follow-up | 12.18 (17.68) | 13.13 (17.66) | -0.16 | -2.31 (-8.44 – 3.82) | -9.74 | 0.461 |
| *HADS-A* |  |  |  |  |  |  |
| Post-Treatment | 6.92 (5.74) | 7.11 (6.15) | 0.44 | -1.16 (-2.89 – 0.57) | -1.32 | 0.187 |
| Follow-up | 7.77 (5.81) | 8.51 (5.65) | 0.33 | -0.61 (-2.34 – 1.13) | -0.69 | 0.492 |
| *HADS-D* |  |  |  |  |  |  |
| Post-Treatment | 6.08 (6.27) | 3.19 (6.20) | 1.05 | -4.49 (-6.36 – -2.63) | -4.72 | <0.001 |
| Follow-up | 5.18 (6.31) | 5.39 (6.34) | 0.39 | -1.40 (-3.27 – 0.47) | -1.47 | 0.142 |
| *EQ-5D* |  |  |  |  |  |  |
| Post-Treatment | 71.34 (28.92) | 71.06 (32.03) | -0.35 | 10.83 (2.57 – 19.10) | 2.57 | 0.010 |
| Follow-up | 67.84 (27.49) | 75.35 (23.23) | -0.82 | 18.54 (10.26 – 26.81) | 4.39 | <0.001 |
| *AAQ-II* |  |  |  |  |  |  |
| Post-Treatment | 35.44 (20.08) | 30.59 (22.76) | 0.79 | -8.65 (-14.33 – -2.97) | -2.98 | 0.003 |
| Follow-up | 28.80 (16.86) | 25.43 (15.83) | 0.66 | -7.16 (-12.85 – -1.47) | -2.47 | 0.014 |

REL: Relaxation; ABCT: Attachment-Based Compassion Therapy; Mn: mean; SD: standard deviation; d: Cohen’s d effect size corrected for repeated measures; B: regression coefficient; 95% CI: 95% confidence interval; Z: value of the Z statistic. p: p-value for each comparison. FIQ: Fibromyalgia Impact Questionnaire; CGI-S: Clinical Global Impression Severity; PCS: Pain Catastrophizing Scale; HADS-A: Hospital Anxiety and Depression Scale-Anxiety; HADS-D: Hospital Anxiety and Depression Scale-Depression; EQ-5D: Visual Analogue Scale of EuroQol; AAQ-II: Acceptance and Action Questionnaire.
